# Flow Synthesis of 2-Methylpyridines via α-Methylation

**DOI:** 10.3390/molecules200915797

**Published:** 2015-08-31

**Authors:** Camille Manansala, Geoffrey K. Tranmer

**Affiliations:** 1 College of Pharmacy, Faculty of Health Science, University of Manitoba, Winnipeg, MB R3E 0T6, Canada; E-Mail: ummanan@myumanitoba.ca; 2 Department of Chemistry, Faculty of Science, University of Manitoba, Winnipeg, MB R3T 2N2, Canada

**Keywords:** continuous flow, 2-methylpyridines, Raney^®^ nickel, green chemistry, α-methylation, Ladenberg rearrangement, flow chemistry

## Abstract

A series of simple 2-methylpyridines were synthesized in an expedited and convenient manner using a simplified bench-top continuous flow setup. The reactions proceeded with a high degree of selectivity, producing α-methylated pyridines in a much greener fashion than is possible using conventional batch reaction protocols. Eight 2-methylated pyridines were produced by progressing starting material through a column packed with Raney^®^ nickel using a low boiling point alcohol (1-propanol) at high temperature. Simple collection and removal of the solvent gave products in very good yields that were suitable for further use without additional work-up or purification. Overall, this continuous flow method represents a synthetically useful protocol that is superior to batch processes in terms of shorter reaction times, increased safety, avoidance of work-up procedures, and reduced waste. A brief discussion of the possible mechanism(s) of the reaction is also presented which involves heterogeneous catalysis and/or a Ladenberg rearrangement, with the proposed methyl source as C1 of the primary alcohol.

## 1. Introduction

As part of a medicinal chemistry project, our group had a need to produce a series of 2-methylpyridines via the α-methylation of substituted pyridines. A quick survey of the literature offered no example of a synthetically useful method that could be performed in less than 2 h using readily available reagents and provide for a 2-methylated pyridine product in a high degree of conversion and regioselectivity. In addition, methylpyridines are valuable chemical products that have been used in a wide range of industries, such as fine chemicals, polymers, agrochemicals, *etc.* [1]. In due course, we set out to develop a robust, bench-top method that would regioselectively provide 2-methylpyridines with a high degree of conversion, and in a synthetically useful yield. To accomplish this task, we resolved ourselves to apply the techniques of continuous flow processing to provide a route to these α-methylated heterocycles in a safe and expedited manner.

Flow chemistry has recently gathered much attention from bench-top synthetic chemists due to its ability to provide synthetic access to desired products in a superior fashion to traditional batch processing, with products fashioned in a much higher degree of efficiency [2]. For instance, several natural products have been produced utilizing flow processing [3,4], including high value pharmaceuticals and complex heterocycles [5]. Several reviews have also been published which describe the many benefits of continuous flow processing [6,7,8,9], and an elaborate explanation of this technique will not be covered here. However, we would like to highlight the fact that flow chemistry replaces conventional fixed-volume reaction vessels with flow reactors that can avoid the need for purification and manipulation of crude reaction products, providing a technique that is shorter and greener [10] than common batch processes. In particular, a common starting material can be passed through a column packed with catalyst or reagent, and a desired product can simply be eluted from the reactor and collected in a flask without the need for further purification or manipulation. Often, these types of reactions can be performed in a relatively short amount of time compared to batch reactions, as a relatively dilute starting material can be passed through a column that is packed with a relatively large amount of catalyst, which quickly produces the desired product as the effective ratio of starting material to catalyst is high. When this process is scaled-out (continuously pumped over a period of time), a significant amount of product can be produced in comparison to the amount of catalyst used, easily surpassing the analogous batch reaction in terms of efficiency [11]. It was our goal to use the many benefits of flow chemistry to rapidly produce a series of 2-methylpyridines in a regioselective manner with a high degree of conversion and yield.

A more thorough examination of the literature details the existing methods that can be used to produce methylpyridines, albeit using batch heterogeneous reactions. Unfortunately, these reactions often require long reaction times, high temperatures, and complex or expensive catalyst systems. One of the problems with existing methods for the synthesis of 2-methylpyridines is the lack of regioselective control and the indiscriminant addition of alkyl groups to the 2, 3, and 4 position of pyridine. For instance, the generation of 2-methylpyridine has been shown to be a byproduct of the oxidation of DMSO/EtOH by H_2_O_2_ mediated by [Fe^II^(O_2_CCF_3_)_2_(py)_4_] in pyridine(py)/trifluoroacetic acid (TFA) under argon, via the formation of pyridine-trapped alkyl radicals. [12] This same group has also previously reported a similar type of reaction, although in this case, the production of 2-methylpyridine was mediated by [Fe^II^(Pic)_2_(py)_4_], a picolinic acid (Pic)-derived catalyst in the presence of DMSO and DMSO/EtOH. [13] All of these reactions proceed via the production of alkyl radicals that add to every position in pyridine, generating 2, 3 and 4-methylpyridines. The take-home message from this method is that generating methyl radicals using these types of catalysts lacks synthetic utility, as generating alkyl radicals in this manner provides insufficient regioselective control. One additional potential method for the synthesis of 2-methylpyridines is the generation of reactive CH_x_ (X = 1–3) species on the surface of a metal catalyst, either through CO or CO_2_ methanation using CO-H_2_ or CO_2_-H_2_. Several examples do exist in the literature, however, the reactions are typically performed in the vapour phase and use specialized catalyst systems. For example, the gas phase methylation of pyridine with CO-H_2_ or CO_2_-H_2_ was performed over a nickel catalyst, producing both 2-picoline (2-methylpyridine) and 2,6-lutidine (2,6-dimethylpyridine) in low conversions and low yields, however, with some degree of regioselectivity *vs*. 3 or 4-methylpyridine (not observed) [14]. In an effort to increase the synthetic utility of these types of reactions, methanol has been used as the alkyl source for the synthesis of methylpyridines; however, these reactions are also performed in the vapour phase. In one example, a series of Ni-Co ferrite catalysts were used for the alkylation of pyridine with methanol using a down-flow vapour-phase reactor that produced 2-, 3-methylpyridines, and 2,6-lutidine as the major products [15]. Once again, these catalysts produced low conversions (17%–50%), with the major product being 3-methylpyridine in most cases. Additionally, a MnFe_2_O_4_ catalyst in a fixed bed reactor gave a moderate product distribution of 68% 2-methylpyridine with 85% conversion (15% pyridine and 17% 3-methylpyridine); however, this reaction was performed at 673 K, and a special down-flow Pyrex glass tubular reactor and setup are required [16].

In terms of synthetic usefulness, one example in the literature did stand out as a method that may suit our purposes and provide a robust route to 2-methylpyridines. Originally, a communication was published in 1964 [17] on the α-methylation of pyridines using Raney^®^ nickel, and a more recent publication reexamined this batch approach in 2008 [18]. Overall, these reactions take pyridine and Raney^®^ nickel in co-operation with a high molecular weight primary alcohol, such as *n*-decyl alcohol (1-decanol) or 1-octanol, which is refluxed at high temperatures (1-decanol BP = 233 °C; 1-octanol BP = 195 °C) for prolonged periods of time. In terms of scale, reactions <150 mmol were performed in 44–68 h, while reactions at preparative scale (~10 mL pyridine) were performed in 212 h, (reaction times up to 438 h were also reported) [18]. One additional problem with this procedure is the need for an acid-wash protocol to separate the α-methylated pyridine from the high-boiling-point solvent, requiring the need for a laborious liquid-liquid extraction procedure. As a result, it was cited in the 2008 publication that “The intrinsically high reaction temperature and long reaction times restrict the application”, with an additional detriment that at preparative scale, 1 g of catalyst is required per 1 mL of pyridine starting material, offering an environmentally unfriendly reaction. As a consequence of these existing methods not possessing the ability to serve our purpose, we set out to overcome some of these synthetic challenges and provide for a robust route to 2-methylpyridines.

## 2. Results and Discussion

Overall, the previous examples highlight the need for the development of a regioselective, synthetically useful method for bench-top organic chemists to reliably generate 2-methylpyridines in good yields. To this end, very few methods exist in the literature for the regioselective α-methylation of pyridine and pyridine derivatives, and we set out to use the many benefits of flow chemistry to solve this problem. Idealistically, due to the heterogeneous nature of the aforementioned reaction, we hoped to pack a column with a portion of Raney^®^ nickel, and use a continuous flow setup to pump a series of pyridines through a column packed with catalyst using a primary alcohol as the solvent. It is our hope that by utilizing this flow process, this reaction will produce α-methylated products in good yields, leading to a synthetically useful procedure which is much greener than the established protocol.

**Figure 1:**
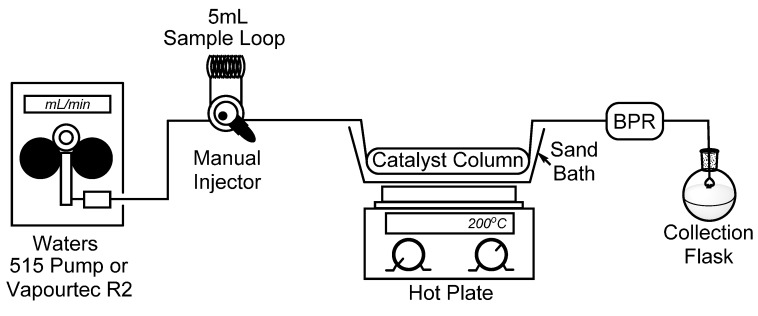
Simple flow chemistry setup for the synthesis of 2-methylpyridines; Waters 515 pump or Vapourtec R2 pumping module, manual dual mode (six-port) injector, steel column packed with Raney^®^ nickel, hot plate with sand bath, and back-pressure regulator.

To begin, we set out to assemble a simplified flow chemistry setup that could be constructed using spare parts which are typically found in a synthetic organic chemistry laboratory. To this end, all that is needed is a pump, a manual HPLC injector with an appropriate sample loop, a metal column to contain the catalyst, and a back-pressure regulator (BPR) to ensure a consistent flow stream. In our bench-top setup, seen in Figure 1, we connected a Waters 515 isocratic HPLC pump to a manual dual mode HPLC sample injector fitted with a 5 mL sample loop which will be used for the introduction of our starting material. It should also be noted that for some of our reactions, the Waters 515 pump was replaced with a Vapourtec R2 pumping module to advance the starting material. Using standard HPLC tubing, we then connected a surplus stainless steel HPLC column (150 × 4.6 mm) which had been cleared of spent stationary phase (~2.5 mL max theoretical volume), followed by a cartridge-type back-pressure regulator. In this case, we chose to use a sand bath and common hotplate as a method to heat our catalyst column, and by covering the sand bath with tinfoil, a consistent temperature of >180 °C was easily maintained. Temperature control, however, may not be ideal using a sand bath, and those seeking more efficient and precise temperature control may choose to utilize alternate methods, such as a gas chromatograph (GC) oven [19].

**Scheme 1:**
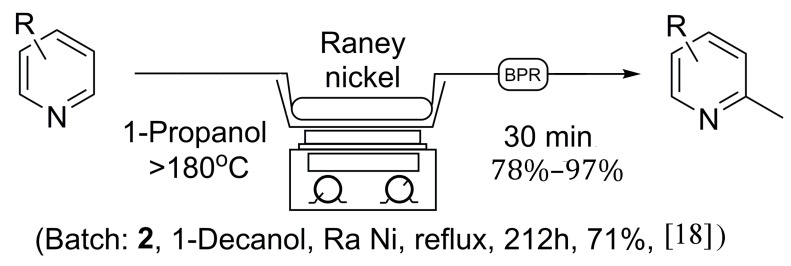
General continuous flow method for the synthesis of 2-methylpyridines in comparison to established batch processing.

Initially, the reaction was first explored using 1-octanol as solvent, as no 1-decanol was available on hand, to ensure the validity of the previous literature examples. In due course, 5.5 grams of Raney^®^ nickel was placed in an empty 150 × 4.6 mm stainless steel column and flushed at room temperature with isopropanol in an attempt to clear water from the catalyst, under which Raney^®^ nickel is normally stored. Once the catalyst column had been prepared, it was placed in-line with our continuous flow system utilizing a 1000 psi back-pressure regulator. A 0.05 M solution of pyridine was prepared in 1-octanol, and injected into the 5 mL sample loop via syringe, while the sand bath was heated to >180 °C as measured by an external thermometer. The pyridine solution was then progressed through the catalyst at a flow rate of 0.1 mL/min, producing 2-methylpyridine at near quantitative conversion, as no pyridine was found to remain by NMR analysis following acid extraction [18]. Overall, this procedure as seen in Scheme 1 holds many advantages to previous batch methods as the reaction time was greatly reduced, and a much safer reaction protocol was discovered. The catalyst column can be reused multiple times, limiting the handling of Raney^®^ nickel and avoiding the need to replenish the catalyst after each reaction. Unfortunately, due to the need for an acid wash procedure to isolate the α-methylated pyridine, the flow protocol using 1-octanol still possesses some limitations.

A well-known benefit of continuous flow processing is its ability to superheat solvents well above the boiling point and perform reactions at elevated temperatures that are not possible for bench-top reflux batch reactions. A back-pressure regulator is essential to maintain a uniform flow stream during this process, and we set out to use a low boiling point alcohol as a replacement for 1-octanol in our continuous flow process. As a result, we set out to optimize our reaction using a similar method as above, instead using 1-propanol as our reaction solvent. 1-propanol has a boiling point of 98 °C, and through the addition of a 1000 psi BPR, we were able to heat the reaction to >180 °C, as measured by an external thermometer in the sand bath which surrounded the column. This solvent switch has a dramatic benefit on the overall reaction as we are able to perform the reaction at a high temperature, and there is no need for an additional acid wash work-up procedure, creating a much more efficient process as we can simply evaporate off the excess alcohol at the end of the reaction. 

In order to find optimized reaction conditions, a series of different starting material concentrations in propanol (0.2, 0.1, 0.05 M) were used in addition to different flow rates from 0.25 to 0.05 mL/min. The final results for the optimized reaction conditions can be seen in Table 1, where seven substituted pyridines and pyridine were passed though the same column packed with Raney^®^ nickel and the solvent evaporated. For our purposes, a concentration of 0.05 M and 0.1 mL/min in 1-propanol appeared to be the optimal reaction condition and the crude product weights over a series of repeated reactions (*n* = 3–5) are shown as the average isolated yield collected over 30 min. In this flow process, simple evaporation of the solvent gave a product that was of sufficient purity for further use by ^1^H-NMR, with some residual 1-propanol remaining in trace amounts. Unfortunately, due to the relatively low boiling points of the products (157 °C for 2,5-lutidine (**2**) *vs.* 98 °C for 1-propanol), extended evaporation of the samples led to lower isolated yields, with the yields listed as the average over multiple runs. In accordance with previous discoveries, the reactions showed a high degree of selectivity and produced products favoring the sterically less hindered α-position, entry **2** and **6**.

**Table 1:** α-Methylation of substituted pyridines.

Entry	Substrate 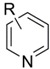	Product 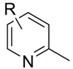	Ave. Isolated Yield (%)
1	Pyridine	2-Methylpyridine	78
2	3-Methylpyridine (3-Picoline)	2,5-Dimethylpyridine	84
3	4-Methylpyridine (4-Picoline)	2,4-Dimethylpyridine	87
4	3,5-Dimethylpyridine (3,5-Lutidine)	2,3,5-Trimethylpyridine	90
5	4- *tert*-Butylpyridine	2-Methyl-4- *tert*-butylpyridine	94
6	3-Phenylpyridine	2-Methyl-3-phenylpyridine	96
7	4-Phenylpyridine	2-Methyl-4-phenylpyridine	97
8	4-(Dimethylamino)pyridine	2-Methyl-4-(dimethylamino)pyridine	91

During the optimization process, it was noted that at higher flow rates and at higher concentrations, some unreacted starting material was observed via ^1^H-NMR. At the slowest flow rate, 0.05 mL/min, and the most dilute conditions (0.05 M), some small amounts of dimethylated product were observed by LC-MS. As a result, a balance is required between the amounts of catalyst needed/residence time, and the concentration/flow rate of the starting material, to ensure complete conversion without over-methylation. It should be noted that the same packed catalyst column was used for each reaction, with >50 reactions being performed for each loading, avoiding the need to replenish the Raney^®^ nickel catalyst after each reaction, offering a much greener and safer reaction than previously reported [18].

In an attempt to determine whether a lower boiling point alcohol could be used in this reaction, and to examine the potential implications on the mechanism of this reaction, a few reactions were performed using methanol as solvent. In this case, however, an additional 500 psi backpressure regulator was required to be placed in series with the previous 1000 psi BPR to ensure a uniform flow stream. Initially, for these methanol examples, the reactions appeared to produce α-methylated products successfully; however, after a few more reactions, the rates of conversion decreased until very little 2-methylated product was produced during subsequent reactions. This result suggests that methanol is incapable of producing the required reactive intermediate on the surface of the catalyst that is required to produce an α-methylated product and a longer-chain primary alcohol is fundamental for this reaction. In this case, once the solvent was switched back to 1-propanol, the α-methylation reactions continued to proceed using the same catalyst column as the unsuccessful methanol reactions. 

**Scheme 2:**
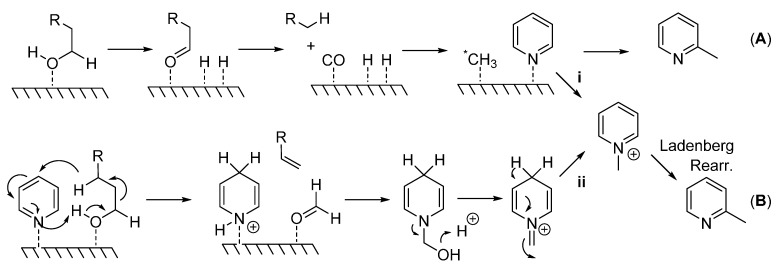
(**A**) Generation of highly reactive methylating species (^*^CH_3_) which attacks pyridine at the sterically less hindered 2-position; (**B**) Generation of pyridinium species which undergoes Ladenberg rearrangement to produce α-methylpyridines.

Although mechanistic studies were not carried out here, Scheme 2 details the potential mechanisms proposed for this type of reaction. A heterogeneous mechanism involves the generation of a methylating species (*CH_3_) that is bound to the surface of the catalyst which attacks the pyridine ring at the sterically less hindered α-position (**A**). In support of this mechanism, the consumption of 1-decanol and the formation of nonane, nonene, carbon monoxide, and dihydrogen [18] suggest C1 as the carbon source and CO as the methyl source via oxidative decomposition of the primary alcohol, path (**A**). Alternately, the formation of an *N*-alkyl pyridinium ion would yield a 2-methylated pyridine species via a Ladenberg rearrangement [15] ((**B**); see also A. Ladenburg *Ber.* **1883**, *16*, 410 and *Ann*. **1888**, *247*, 1). In order for this reaction to occur, either a pyridinium species is directly generated from a reactive methylating species (**i**), or dihydropyridine, an alkene, and a formyl group (HCOH) is produced. Formaldehyde then reacts with dihydropyridine to form the pyridinium species (**ii**) which undergoes the Ladenberg rearrangement under high temperatures. The formation of nonane and nonene previously reported would also allow for this type of mechanism [18].

## 3. Experimental Section

All chemicals were purchased from Sigma-Aldrich Co. (St. Louis, MO, USA) and used without further purification, including Raney^®^ nickel (Prod. #221678, Lot #MKBK4869V). All products are known compounds and identified by LC-MS and/or ^1^H-NMR spectroscopy and were found to be in accordance with literature values. All ^1^H-NMR were recorded on a Bruker Avance 400 spectrometer (Fremont, CA, USA) in CDCl_3_ as internal standard. LC-MS were performed in the ESI mode using a Waters 2795 HPLC (Milford, MA, USA) coupled to a Micromass ZQ single quadrupole MS.

### 3.1. Representative Batch Procedure for the Synthesis of 2-Methylpyridines 

The procedure was performed in accordance with known literature examples [18]. Approximately 1 g of Raney^®^ nickel was placed in a 50 mL round bottom flask and washed with two portions of ethanol (2 × 15 mL) followed by dilution with 25 mL of 1-octanol. Then 4-Phenylpyridine (1.0 g, 6.44 mmol) was placed in the flask and refluxed for 19 h (~200 °C). The flask was cooled, and the crude reaction was filtered through a small plug of celite, and the filtrate was washed with 1 N HCl (4 × 25 mL). The combined aqueous layers were then made basic with 2 M NaOH solution and extracted with diethyl ether (4 × 25 mL). The combined organic extracts were then subjected to rotary evaporation and concentrated to give a mixture of 2-methyl-4-phenylpyridine, and unreacted starting material by ^1^H-NMR (0.73 g, 67% crude yield).

### 3.2. General Flow Procedure for the Synthesis of 2-Methylpyridines

The continuous flow system was set up as described in Figure 1 by using either a Waters 515 pump or Vapourtec R2 pumping module to progress 1-propanol through a stainless steel column (150 × 4.6 mm) packed with 5.5 g of Raney^®^ nickel. Initially, while a 0.05 M solution of the respective pyridine was prepared in 5 mL of 1-propanol, the flow rate was set to 0.3 mL/min and the catalyst column was heated in a sand bath to >180 °C for 30 min. The flow rate was then set to 0.1 mL/min and the sample was placed in a 5 mL sample loop and introduced into the reaction stream. The dead volume had been estimated at ~1 mL (~2.5 mL max theoretical, 150 × 4.6mm cylinder) giving a ~10 min residence time: however, the sample was collected for 30 min to ensure complete collection. The solvent was evaporated to yield a product that was of sufficient purity for further use. 

For all of the products, the analytical data for each molecule was in accordance to that previously reported [18] and additional references can be found accordingly as indicated: 2-methylpyridine [20]; 2,5-dimethylpyridine [21]; 2,4-dimethylpyridine [22]; 2-methyl-4-*tert*-butylpyridine [23]; 2-methyl-3-phenylpyridine [24]; 2-methyl-4-phenylpyridine [25]; 2-methyl-4-(dimethylamino)pyridine [26].

## 4. Conclusions

As highlighted previously, we set out to develop an expedited, synthetically useful protocol for the synthesis of simple 2-methylpyridines using a continuous flow process. As a result, using a simplified bench-top flow chemistry setup, we were able to pack a surplus HPLC column with Raney^®^ nickel and, using a pumping module, progress a series of pyridines through our heterogeneous catalyst and produce α-methylated products with a high degree of selectivity. The key advantages of this protocol were in its ability to provide for shorter reaction times, increased safety, avoidance of work-up procedures, and reduced waste in comparison to batch methods. These benefits were realized through the use of a low boiling point alcohol heated to high temperatures that produced 2-methylpyridines in very good isolated yields which were suitable for further use without additional purification. Additional contemplation on the potential mechanism(s) pointed towards the generation of an activated methylation group and/or Ladenberg rearrangement, giving the high degree of selectivity observed. 
